# Small Messengers: Glioblastoma-Derived Extracellular Vesicles Modulate γδ T Lymphocytes Through a MIC-Dependent Mechanism

**DOI:** 10.3390/biology15030275

**Published:** 2026-02-03

**Authors:** Micaela Rosato, Paula Saibene Vélez, Alejandra Infante Cruz, Matías A. Pibuel, Federico Fuentes, Mónica Vermeulen, Juan Iturrizaga, Pablo E. Espil, Silvia Berner, Gabriela V. Salamone, Carolina C. Jancic

**Affiliations:** 1 Instituto de Medicina Experimental–CONICET–Academia Nacional de Medicina, Pacheco de Melo 3081, Buenos Aires 1425, Argentinapaula.saibene@gmail.com (P.S.V.);; 2 Departamento de Química Biológica, Facultad de Ciencias Exactas y Naturales, Universidad de Buenos Aires, Buenos Aires 1428, Argentina; 3 Instituto de Estudios de la Inmunidad Humoral (IDEHU)–CONICET, Departamento de Microbiología, Inmunología y Biotecnología, Facultad de Farmacia y Bioquímica, Universidad de Buenos Aires, Buenos Aires 1113, Argentina; 4 Departamento de Microbiología, Parasitología e Inmunología, Facultad de Medicina, Universidad de Buenos Aires, Buenos Aires 1121, Argentina; 5 División Neurocirugía, Instituto de Investigaciones Médicas A. Lanari, Universidad de Buenos Aires, Buenos Aires 1427, Argentina; 6 Servicio de Neurocirugía, Clínica y Maternidad Santa Isabel, Buenos Aires 1406, Argentina; pabloezequielespil@gmail.com (P.E.E.);

**Keywords:** γδ T cells, glioblastoma, extracellular vesicles

## Abstract

Glioblastoma (GBM) is the most aggressive and common primary brain tumor, with a median survival of less than a year after diagnosis. γδ T lymphocytes are immune cells that can migrate to tumors and induce malignant cells’ apoptosis. Our previous in silico studies showed that higher γδ T-cell infiltration in GBM correlates with better patient survival, and in vitro experiments showed that GMB supernatants promote an antitumoral profile in γδ T cells. Extracellular vesicles (EVs) play a critical role in cell communication, particularly in tumor microenvironment modulation. Here, we studied the mechanisms responsible for γδ T lymphocyte activation by GBM-derived EVs, together with the effect of these EVs on γδ T cells from GBM patients. For that, γδ T cells were purified from peripheral blood, and EVs were obtained from U251 cell supernatants by differential centrifugation. After EV characterization, we evaluated the γδ T cell–EV interaction and γδ T-cell modulation by EVs. Results showed that EVs induced an increase in CD69 expression, cytotoxicity, and TNF-α and IFN-γ production in γδ T cells in a MIC-dependent mechanism. These results provide valuable insights for developing targeted immunotherapies in GBM patients.

## Simple Summary

Glioblastoma is an aggressive brain tumor that evades the immune system; this study asked whether tiny particles released by glioblastoma cells (extracellular vesicles, or EVs) can influence a specific type of immune cell called a γδ T lymphocyte. For that purpose, we first characterize EVs from the U251 glioblastoma cell line and test whether they bind to, enter, and activate γδ T cells, and which molecules on EVs drive that effect. We found that the isolated EVs were enriched in medium/large sizes, contained tumor markers including EGFR and the stress ligands MICA/B, and were taken up by γδ T cells. Exposure to these EVs increased an early activation marker (CD69), raised production of inflammatory proteins TNF-α and IFN-γ, and enhanced the T cells’ ability to kill tumor cells. Blocking MICA/B on EVs reduced activation. These results suggest U251-derived EVs can stimulate antitumor γδ T-cell responses via MICA/B, offering a potential avenue to boost immune therapies and improve outcomes for patients.

## 1. Introduction

Glioblastoma (GBM) is an adult-type diffuse glioma and the most frequent and aggressive primary brain tumor. It is defined by rapid progression and an immunosuppressive microenvironment that undermines effective antitumor immunity. Histopathologically, GBM is characterized by tissue necrosis and abnormal endothelial proliferation, features that contribute to its exceptionally high lethality [1,2,3]. As GBM cells proliferate rapidly and infiltrate diffusely into adjacent healthy brain tissue, they make it difficult to achieve complete remission and a median survival of less than one year after diagnosis [4]. At present, GBM treatment relies primarily on surgical removal of the tumor, typically followed by the conventional treatment including radiation therapy, temozolomide, and bevacizumab, which gives modest benefit. Emerging strategies aim to improve outcomes by combining immunotherapies, advanced drug delivery, gene editing technologies, and targeted inhibitors [2,5]. Despite advances in diagnostic imaging and therapeutic strategies, these approaches provide only temporary and limited benefits [6], and clinical outcomes remain unfavorable for highly malignant and invasive forms [7,8,9]. More recently, cancer immunotherapy, successful in extracranial malignancies such as melanoma, non-small-cell lung cancer, and non-Hodgkin lymphoma, has been explored in GBM. The disappointing results of clinical trials highlight the urgent need for novel therapeutic strategies [10].

Intercellular communication within the tumor microenvironment is a major determinant of immune evasion and therapeutic resistance, and tumor-derived extracellular vesicles (EVs) have emerged as potent modulators of the immune landscape within the tumor microenvironment. EVs are released by all cellular organisms [11] and are classified according to their cellular origin, biogenesis pathways, and cargo composition [12]. Two principal EV groups are widely recognized: small EVs, which mainly originate as intraluminal vesicles within endosomal multivesicular bodies that fuse with the plasma membrane to release their contents, and their size ranges from 30 to 100 nm, and medium/large EVs, which form by direct outward budding and fission of the plasma membrane and measure 100–1000 nm. The distinct biogenesis routes underlie differences in EV composition and function, with important implications for intercellular signaling and disease biology [11]. Moreover, EVs constitute a widespread intercellular communication system operating across diverse cell types and pathological contexts. They either mediate biological effects after uptake by recipient cells or through direct engagement of EVs’ surface ligands with receptors and membrane components on target cells [13,14,15,16]. Dysregulated release of specific EV subpopulations has been implicated in numerous diseases, including cancer, neurological disorders, and immune pathologies, where EV-driven signaling can reprogram recipient cells and promote disease progression [15,16]. EVs carry a complex and functionally diverse cargo: proteins (including cytokines, chemokines, and regulators of signaling and migration), lipids, glycoconjugates, and nucleic acids such as mRNAs and microRNAs [17], which enables them to modify cells both within the tumor microenvironment and at distant sites. By delivering these molecular signals, tumor-derived EVs reshape innate and adaptive immunity, driving natural killer cells (NK), CD4^+^ and CD8^+^ T cells, dendritic cells, macrophages, and myeloid-derived suppressor cells toward immunosuppressive phenotypes [18]. EV surfaces also can display key immunoregulatory molecules, such as CTLA–4, PDL–1, and FASL [19,20], as well as the ectoenzymes CD39 and CD73 [21]; thus, they can convey stress-induced ligands and other modulators of immune response, thereby contributing to immune escape and therapeutic resistance, collectively reinforcing immune evasion.

Of note, human GBM cells exhibit markedly increased EV release in vivo [15]. GBM-derived EVs have been shown to influence tumor invasion, angiogenesis, and immune modulation, acting as small messengers that reshape the tumor microenvironment and alter immune cell phenotypes and functions [22].

Within the GBM tumor microenvironment, the infiltrating immune populations are diverse; there are tumor-associated macrophages, immunosuppressive myeloid-derived suppressor cells, and regulatory T cells. These cell types establish a suppressive milieu that fosters tumor growth and undermines cytotoxic T-cell activity [23]. Despite their reduced frequency among GBM-infiltrating leukocytes, T lymphocytes constitute a pivotal component of antitumor immunity and are therefore critical targets for therapeutic modulation [22]. Interestingly, γδ T cells are non-conventional T lymphocytes with potent cytotoxic activity against transformed cells and show a capacity to respond to stress-induced ligands and to migrate into tumors [24]. γδ T lymphocytes, originally described in 1986 [25], arise during ontogeny from double-negative thymocytes (CD4^−^CD8^−^) and express a clonally distributed T-cell receptor (TCR) composed of γ and δ chains with a relatively restricted repertoire [26,27]. In peripheral blood, the Vγ9Vδ2 subset accounts for approximately 5–10% of CD3^+^ cells and represents the predominant circulating γδ population in healthy humans [28]. Unlike conventional αβ T cells, γδ T cells do not rely on classical peptide presentation by MHC molecules; this MHC-independent recognition underlies their potential as candidates for antitumor immunotherapy [29]. γδ T cells exert tumor immunosurveillance by detecting stressed or transformed cells. A central activating axis for this process occurs, which involves the activating receptors shared with NK cells, such as the NKG2D receptor, which recognizes the stress-induced ligands MICA/MICB [30,31]. Notably, GBM cells express several stress-associated, MHC class I-like ligands, including MICA/B, that can be engaged by γδ T cells via NKG2D or the TCR, facilitating recognition of malignant cells [30,31,32]. Since γδ T cells do not depend on classical peptide presentation by MHC molecules, they can target tumors in an MHC-independent manner, and this distinctive recognition mode underpins their promise as candidates for antitumor cell therapy [29,33]. Thus, in the last few years, adoptive transfer of γδ T lymphocytes has arisen as an immunotherapeutic strategy for GBM. As noted above, GBM-derived EVs can reshape the tumor microenvironment; consequently, they may directly perturb γδ T-cell behavior and thereby facilitate immune evasion by GBM. Although accumulating evidence indicates that GBM EVs modulate various immune populations, the specific effects of GBM-derived EVs on γδ T-cell activation, receptor engagement, and effector function remain poorly defined. We therefore hypothesize that GBM EVs modulate γδ T-lymphocyte responses by modifying ligand availability and receptor signaling. Demonstrating such a mechanism would directly connect EV-mediated intercellular communication to a defined immune recognition pathway and could reveal novel targets to restore or enhance γδ T-cell-mediated antitumor activity in GBM.

## 2. Materials and Methods

The experimental protocols performed were approved by the Biosafety and Research Review Board of Instituto de Medicina Experimental—CONICET—Academia Nacional de Medicina and the Ethical Committee of the Institutos of the Academia Nacional de Medicina. The methods were carried out following the approved guidelines. The number of samples from healthy donors employed in this study was 22, and for GBM patients, it was 4 (Supplementary Table S1).

Reagents and antibodies: Ficoll–Hypaque was from GE Healthcare Bio-Sciences AB (Uppsala, Sweden). Anti-TCR γδ MicroBead kit was obtained from Miltenyi Biotec (Bergisch Gladbach, Germany). The fetal bovine serum (FBS) was from Invitrogen (Carlsbad, CA, USA). Dulbecco’s Modified Eagle’s Medium (DMEM), Trypsin–EDTA, sodium pyruvate, L-glutamine, and penicillin/streptomycin were from Gibco, Thermo Fisher Scientific (Waltham, MA, USA). PE mouse anti-human CD69, CD63, TCRγδ, EGFR, and MICA/B antibodies; PerCP mouse anti-human CD69 and CD45, PE/Cy7 mouse anti-human PD–1, APC mouse anti-human CD63, FITC mouse anti-human CD81, PerCP/Cy5.5 mouse anti-human CD9 antibodies, purified and biotin mouse anti-human TNF-α and IFN-γ antibodies; and purified mouse anti-human MIC and PD–L1 antibodies were from BioLegend (San Diego, CA, USA). PE mouse anti-human PD-L1 was from eBioscience, Thermo Fisher Scientific (San Diego, CA, USA). Mouse anti-human purified CD63 was from BD Pharmingen (Franklin Lakes, NJ, USA). HMBPP was obtained from Cayman Chemical (Ann Arbor, MI, USA). PKH26 was from Sigma-Aldrich (St. Louis, MO, USA). TRIzol was from Thermo Fisher Scientific (Waltham, MA, USA).

Peripheral blood γδ T-lymphocyte isolation: γδ T cells were isolated from heparinized human blood from healthy donors and GBM patients, who gave written informed consent, by centrifugation on Ficoll–Hypaque, with positive selection carried out using magnetic microspheres covered with anti-TCR γδ antibodies, according to the manufacturer’s instructions (Miltenyi Biotec, Order No. 130-050-701). Briefly, 10^7^ cells were resuspended in 40 μL of PBS containing 0.5% BSA and 2mM EDTA (staining buffer) and 5 μL of anti-TCR γ/δ hapten antibody. Cells were mixed and incubated for 10 min at 4–8 °C. Afterward, 30 μL of buffer and 10 μL of MACS anti-hapten FITC conjugated MicroBeads were added per 10^7^ total cells. After 15 min at 4–8 °C, cells were washed in staining buffer and centrifuged. Afterward, cells were resuspended in 500 μL of the same buffer and proceeded to magnetic separation. After purification, cells were resuspended in DMEM supplemented with 10% heat-inactivated FBS and penicillin (100 U/mL)/streptomycin (100 µg/mL) (supplemented DMEM) and used to perform the assays within one hour after isolation. Cells were analyzed by flow cytometer (FACSCalibur, Becton Dickinson, San Jose, CA, USA) to guarantee that γδ T-cell purity was >98% and monocyte contamination was <2%, as previously described [34,35,36]. The purification procedure did not affect cell activation.

U251 cell culture: GBM cell line U251 was cultured in DMEM supplemented with 10% of heat-inactivated FBS, L-glutamine (2 mM), sodium pyruvate (1 mM), and penicillin (100 U/mL)/streptomycin (100 µg/mL) at 37 °C with 5% CO_2_ atmosphere in 75 cm^2^ culture flask for large-scale expansion.

GBM sample disaggregation: The samples were obtained after surgical removal of the tumor from a patient with GBM, who gave written informed consent at the División Neurocirugía, Instituto de Investigaciones Médicas, A. Lanari or at Clínica y Maternidad Santa Isabel. The biopsy was incubated with collagenase (2 mg/mL) and DNAse (1000 IU) for 30 min at 37 °C, then inactivated with 10% FBS and 2 mM EDTA. Afterward, the cells were recovered and stained for CD45 and TCRγδ according to standard protocols and analyzed by flow cytometry.

GBM-derived EV separation: GBM-derived EVs were obtained from confluent monolayers of U251 cell line. When cells were in 80–90% of confluence, their medium was replaced by supplemented DMEM previously depleted from EVs (centrifuged at 100,000× *g*, overnight (ON)). Then, 24 h later, the cell lines’ conditioned medium was collected, and EVs were obtained following the protocol described by Fraser et al. [37] Briefly, the conditioned medium was centrifuged at 300× *g* for 5 min at 4 °C. The resulting supernatant was collected in a 15 mL tube and then centrifuged at 2000× *g* for 10 min at 4 °C to discard cell debris. The obtained supernatant was collected in microtubes and centrifuged at 10,000× *g* for 30 min at 4 °C. Finally, supernatants were discarded, pellets were recovered and washed with PBS (previously filtered with 200 nm filters), and then resuspended in depleted supplemented DMEM or PBS and stored at −80 °C. Meanwhile, U251 cells were trypsinized and counted in order to determine the number of source cells for the EV sample.

Transmission electron microscopy (TEM) of GBM-derived EVs: Samples were negatively stained for electron microscopy following the protocol described by Théry et al. [38]. Briefly, samples obtained at 10,000× *g* were fixed with paraformaldehyde (PFA) 2% and then applied to Formvar–carbon-coated grids and allowed to adsorb for 20 min in a dry environment. Grids were subsequently washed with PBS, and fixation was completed with an incubation in 1% glutaraldehyde (EMS, Hatfield, PA, USA) for 5 min. After eight washes in distilled water, grids were first incubated for 5 min in neutral uranyl oxalate, then transferred to a mixture of 4% uranyl acetate and 2% methyl cellulose for 10 min. Excess embedding medium was carefully blotted to generate a thin and uniform methyl-cellulose film, and then grids were air-dried for 5–10 min and stored until imaging. Samples were examined by TEM with a LEO–906 microscope (ZEISS, Oberkochen, Baden–Württemberg, Germany) at 80 kV.

Nanoparticle tracking analysis (NTA) of GBM-derived EVs: NTA and zeta (z) potential measurements were performed using a ZetaView PMX–120 instrument (Particle Metrix GmbH, Inning am Ammersee, Germany), software v8.06.01 with a 488 nm laser. Samples obtained at 10,000 × *g* were resuspended in 20 µL of filtered PBS and stored at −80 °C. Samples were thawed and diluted in PBS until optimal concentrations for measurements were achieved. Analyses were performed at 25 °C and pH = 7, with the instrument set to sensitivity = 80, shutter = 100, and frame rate = 30 fps. Particle size distribution and concentration were determined by performing one measurement cycle across 11 distinct focal positions, while the z potential measurements were done in a stationary mode with continuous tracking over two measurement cycles.

Flow cytometry of GBM-derived EVs: Samples obtained at 10,000× *g* were washed with 100 µL of PBS and stained with DAPI or fluorochrome-conjugated antibodies in staining buffer (5 µL) for 30 min at 4 °C. Afterwards, samples were washed with 100 µL of staining buffer (10,000× *g*, 30 min) and fixed in 100 µL of PFA 1% for 15 min at room temperature (RT). They were then washed with 100 µL of PBS and stored ON at 4 °C prior to flow cytometry analysis. All buffers were filtered with 200 nm filters before use. Samples were acquired using a Cytek Northern Lights 3000 full-spectrum flow cytometer (Cytek Biosciences, Fremont, CA, USA).

To confirm that fluorescent signals originate from EVs, assay controls were included, as described by Welsh et al. [39] (Supplementary Figure S1). To differentiate EVs from contaminant particles and antibody aggregates, a buffer with reagent control was used. Detergent treatment with Triton X–100 was included to verify that signals originate from membrane-enclosed particles.

Quantification of RNA from GBM-derived EVs: EVs obtained from U251 cells were lysed with TRIzol and stored at –20 °C ON. Chloroform was added to the suspension, and it was incubated for 5 min before centrifugation at 12,000× *g* (15 min, 4 °C). The topper phase was recovered in a new tube, and RNA was precipitated by adding isopropanol, incubating for 10 min at RT, and centrifugating at 12,000× *g* (10 min, 4 °C). The resulting supernatant was discarded, and the pellet was resuspended in ethanol 75% and then centrifuged at 7500× *g* (5 min, 4 °C). The supernatant was again discarded, and the pellet was left to dry for 10–15 min. Finally, RNA samples were resuspended in 20 µL of RNAse-free distilled water and incubated at 60 °C for 10 min. They were quantified with a DS–11 Series UV–visible spectrophotometer (DeNovix, Wilmington, DE, USA).

Western blotting: Samples were lysed with Tris–HCl (60 mM, pH 6.8)/SDS 1% using EDTA-free protease inhibitor cocktail (Thermo Scientific, Waltham, MA, USA). A total of 15 μg of protein samples—quantified by UV–visible spectrophotometry—were loaded onto 12% SDS–PAGE under non-reducing conditions and transferred to a polyvinylidene difluoride membrane (Amersham Hybond-P. Cytiva, Marlborough, MA, USA). Membranes were blocked in PBS with 0.1% BSA, 0.4% Tween, and 1 mM EDTA for 1 h at RT and incubated with primary anti-human CD63 antibodies (BD Pharmingen, Franklin Lakes, NJ, USA) (2 μg/mL, ON, 4 °C). Afterwards, they were incubated with anti-species secondary antibodies conjugated to HRP (horseradish peroxidase) during 4 h at RT. Blots were detected by chemiluminescence using a solution containing luminol and hydrogen peroxide.

Interaction studies: EVs obtained from 6 × 10^6^ U251 cells were labeled with the lipophilic fluorescent dye PKH26 (2 µM) following the manufacturer’s instructions, with the modification that centrifugation steps were performed at 10,000× *g* for 30 min at 4 °C. Purified γδ T cells were activated or not with HMBPP (1 µM for 1 h at 37 °C), and then 6 × 10^4^ cells were incubated during 3 h or ON with labeled EVs in depleted medium in U-bottom 96-well plates. After incubation, γδ T cells were collected and stained with 4′,6-diamidino-2-phenylindole (DAPI: 1 µ/mL) for nucleus visualization and then observed with a fluorescent confocal microscope (Olympus FV–1000 (Tokyo, Japan)). Twenty 60X images per condition were analyzed with ImageJ 1.52n software (National Institute of Health, USA). Z-stack acquisition was performed to capture three-dimensional interactions.

Treatment of γδ T cells with GBM-derived EVs: First, 6 × 10^4^ purified γδ T cells were centrifuged (2600× *g*, 2 min), resuspended in vesicles in depleted supplemented DMEM, and cultured with or without U251-derived EVs in U-bottom 96-well plates. EV samples from different amounts of U251 cells (2–8 × 10^6^) were tested. After ON incubation at 37 °C, γδ T cells were recovered for immunostaining and supernatants for ELISA analysis.

Cells immunostaining and flow cytometry: Recovered γδ T cells from previously described assays were incubated with saturating concentrations of anti-CD69 and anti-PD–1 monoclonal mouse antibodies in staining buffer for 30 min at 4 °C. After incubation, cells were washed with 300 µL of the buffer and then with PBS by centrifugation at 2600× *g* for 2 min. Supernatants were discarded, pellets were fixed with PFA 1%, and samples were analyzed with a FACSCalibur flow cytometer (Becton Dickinson, San Jose, CA, USA).

Cytokine production assay: IFN-γ and TNF-α production by cells were quantified in the supernatants recovered from the previously described assays by ELISA following conventional protocols provided by the manufacturer.

Cytotoxicity assay: First, 5 × 10^4^ U251 cells were seeded in 12-well culture plates. After ON incubation, purified γδ T cells were co-cultured with U251 cell line monolayers in a 1:1 ratio, with or without U251-derived EVs. All conditions were performed in vesicle-depleted supplemented DMEM. As a control, γδ T cells were cultured alone. After 48 h incubation at 37 °C, cells were trypsinized and stained with propidium iodide (PI) (1 µg/mL) for flow cytometry analysis. The percentage of PI+ cells was determined, excluding lymphocyte population, in order to analyze U251 cells.

Blocking assays: EVs obtained from 6 × 10^6^ U251 cells were incubated for 1 h with purified anti-human MIC or PD–L1 antibodies (10 µg/mL). Afterwards, EVs and ligand-blocking antibodies were added to cultures of 6 × 10^4^ γδ T cells and left to incubate ON. Mouse IgG antibody (10 µg/mL) was used as control. γδ T cells’ activation was assessed by CD69 immunostaining and flow cytometry.

Informatics and statistical analysis: For experimental approaches, statistical analysis was performed using GraphPad Prism 8.0.2 for Windows, GraphPad Software (La Jolla, CA, USA). Statistical significance was defined as *p* < 0.05.

To perform in silico studies, data repository from The Cancer Genome Atlas (TCGA) was used and then analyzed using TIMER3.0 software https://compbio.cn/timer3/ (accessed on 17 December 2025) [40]. TIMER3.0 employed RNA–Seq expression data of *TRGV9*, *KLRK1*, *PDCD1*, *EGFR*, *MICA/B*, and *CD274* from GBM samples (n = 287).

## 3. Results

### 3.1. Characterization of EVs Derived from GBM Cell Line U251

**Figure 1:**
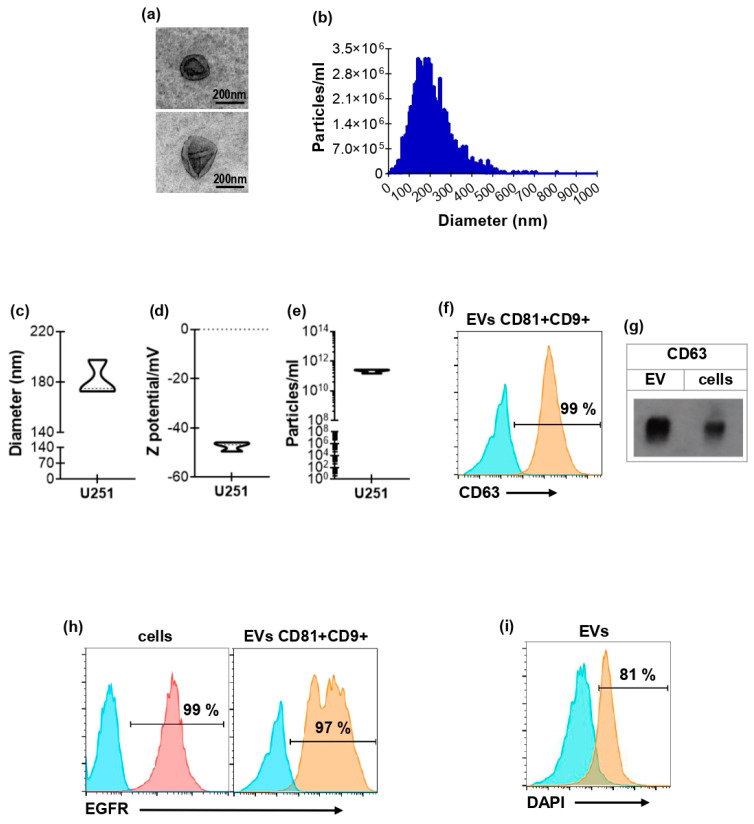
Characterization of EVs derived from GBM cell line U251. (**a**) Representative images, obtained by transmission electron microscopy, of EVs derived from U251 cells. Scale bar = 200 nm. (**b**) Representative histogram of U251 EVs’ size distribution in one sample, measured by NTA. Mean diameter (**c**), Z potential (**d**), and concentration values (**e**) of U251 EV samples measured by NTA (n = 4). (**f**) Representative histogram showing the percentage of CD9+/CD81+ U251 EVs expressing CD63, measured by flow cytometry. Blue histogram represent FMO and the orange EVs staining. (**g**) Representative Western blot for CD63 expression in both U251 cells and EVs. (**h**,**i**) Representative histograms obtained by spectral flow cytometry showing the percentage of U251 cells (**h**, **left panel**) and CD9+/CD81+ U251 EVs (**h**, **right panel**) expressing EGFR. (**i**) Representative histogram of DAPI+ EVs. Blue histograms represent FMO, pink represent cell staining and orange EVs staining.

The material isolated from the supernatants of the GBM cell line U251 via differential centrifugation was analyzed to determine the presence of structures compatible with EVs [41]. To this end, we evaluated morphology using TEM; size, solubility, and concentration via NTA; and the presence of EV markers through spectral flow cytometry and Western blot analysis. As shown in Figure 1a, the isolated material exhibits a well-defined structure, with morphology and size compatible with an enriched population of medium/large EVs, as expected given the employed isolation methodology. The NTA analysis showed a size of 184.0 ± 6.1 nm (Figure 1b,c), which correlates with TEM observations. Additionally, we assessed the zeta potential (Figure 1d and Table 1), which indicates the strength of the electrostatic repulsion between particles; values more negative than −30 mV are generally associated with a lower risk of aggregation. Finally, we determined the concentration by NTA that was 2.1 × 10^11^ ± 0.3 × 10^11^/mL (Figure 1e and Table 1). Moreover, the particles express EV-associated tetraspanins CD9 and CD81, along with the classical marker CD63, as confirmed by both spectral flow cytometry (Figure 1f) and Western blot (Figure 1g). It is well known that EVs reflect the characteristics of their precursor cells. Interestingly, the material obtained in this work expresses the epidermal growth factor receptor (EGFR) (Figure 1h), a recognized GBM tumor marker [37]. We also confirmed the presence of nucleic acids within the vesicles through DAPI staining (Figure 1i), and RNA quantification by UV–visible spectrophotometry (Table 1). Taken together, these results allow us to classify the material obtained from the U251 cell line as medium/large EVs.

### 3.2. GBM-Derived EVs Interact with γδ T Cells

**Figure 2:**
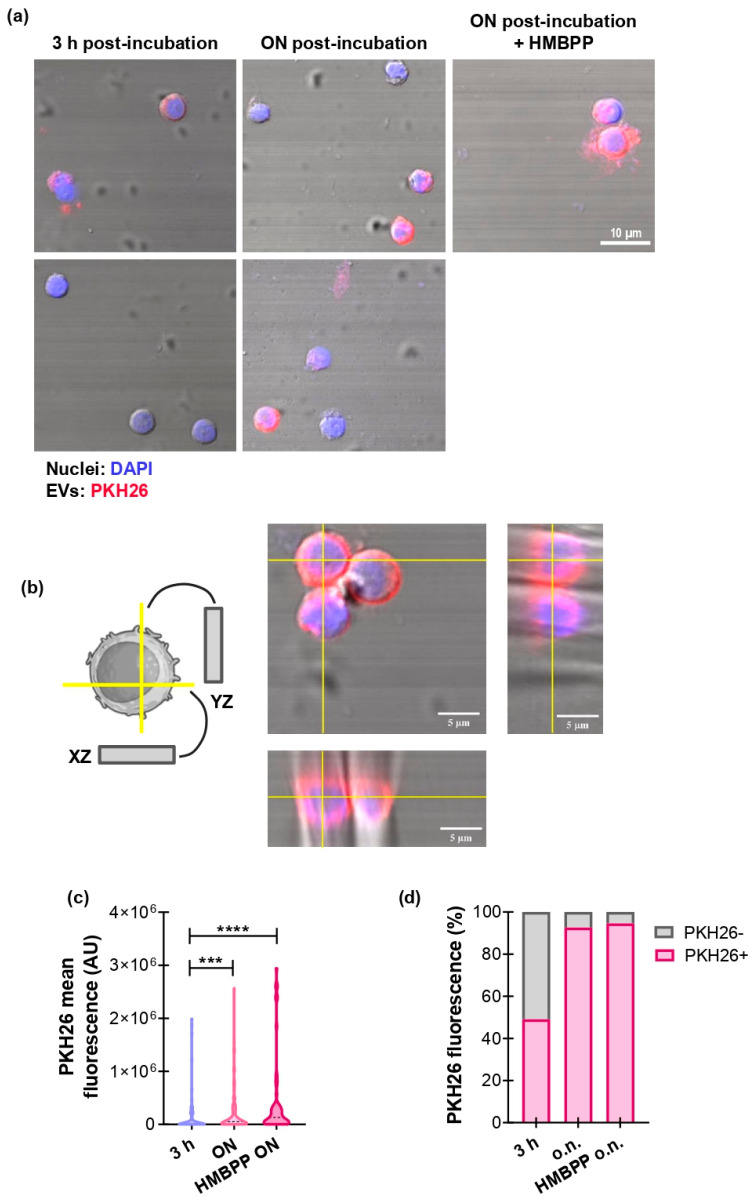
GBM-derived EVs interact with γδ T cells. (**a**–**d**) After purification, γδ T cells were activated or not with HMBPP 1 µM and incubated for 3 h or ON with U251-derived EVs previously stained with PKH26 (red). γδ T cells were then collected and observed by confocal microscopy. (**a**) Representative images of PKH26+ γδ T cells. Nuclei were stained with DAPI (blue). Magnification: 60X. (**b**) Diagram (**left panel**) and representative images (**right panel**) of orthogonal views of a z-stack of one single cell of HMBPP in the ON condition. (**c**) Mean color intensity of PKH26 in γδ T cells. *** *p* < 0.001, **** *p* < 0.0001, Kruskal–Wallis and Dunn’s multiple comparisons test. (**d**) Percentage of PKH26+ and PKH26– γδ T cells for each condition.

To explore whether GBM-derived EVs engage γδ T cells, we performed confocal microscopy analysis of co-cultures and quantified the frequency of cell–EV contact, their spatial distribution, and the kinetics of EV internalization (Figure 2). For that purpose, EVs were fluorescently labeled with PKH26 and added to primary human γδ T cells for 3 h or ON (with or without HMBPP). Confocal images reveal frequent close location of PKH26+ EVs to γδ T-cell membranes, with vesicles appearing to overlap the plasma membrane and extend into the cytoplasm (Figure 2a). EVs were observed in distinct morphological states consistent with sequential steps of engagement: surface adhesion, uptake, and intracellular localization. Orthogonal projections of z-stacks confirmed that PKH26 signal localized beneath the cell surface, supporting bona fide internalization rather than surface-restricted fluorescence (Figure 2b). Quantitative readouts corroborated the imaging observations. Moreover, PKH26 mean fluorescence intensity per cell increased progressively over time (Figure 2c), indicating accumulation of EV-derived material. The percentage of PKH26-positive γδ T cells increased from baseline to approximately 50% at 3 h and continued to increase at later time points until the majority of cells were positive (Figure 2d). Similar results were observed in γδ T-cell activation, whether or not with the specific agonist HMBPP. Together, these results indicate that the PKH26 signal reflects genuine EV–cell interactions and uptake.

### 3.3. GBM-Derived EVs Activate γδ T Cells

The observed EVs and γδ T-cell contact and subsequent internalization prompted us to evaluate whether this interaction was associated with γδ T-cell activation; accordingly, we measured activation markers and cytokine production by cells after EV exposure. For that purpose, freshly isolated γδ T lymphocytes obtained from the peripheral blood of donors were cultured with EVs derived from the GBM cell line U251. First, to determine the quantity of EVs to be employed in those experiments, we perform an EV titration in which γδ T cells were incubated with EVs obtained from different numbers of U251 cells. We found that the statistically significant modulation of γδ T cells by EVs was observed when using EVs obtained from 6 × 10^6^ cells (Supplementary Figure S2), equivalent to an absolute particle number of 4.15 × 10^9^ ± 0.57 × 10^9^ (calculated from NTA results, Table 1).

**Figure 3:**
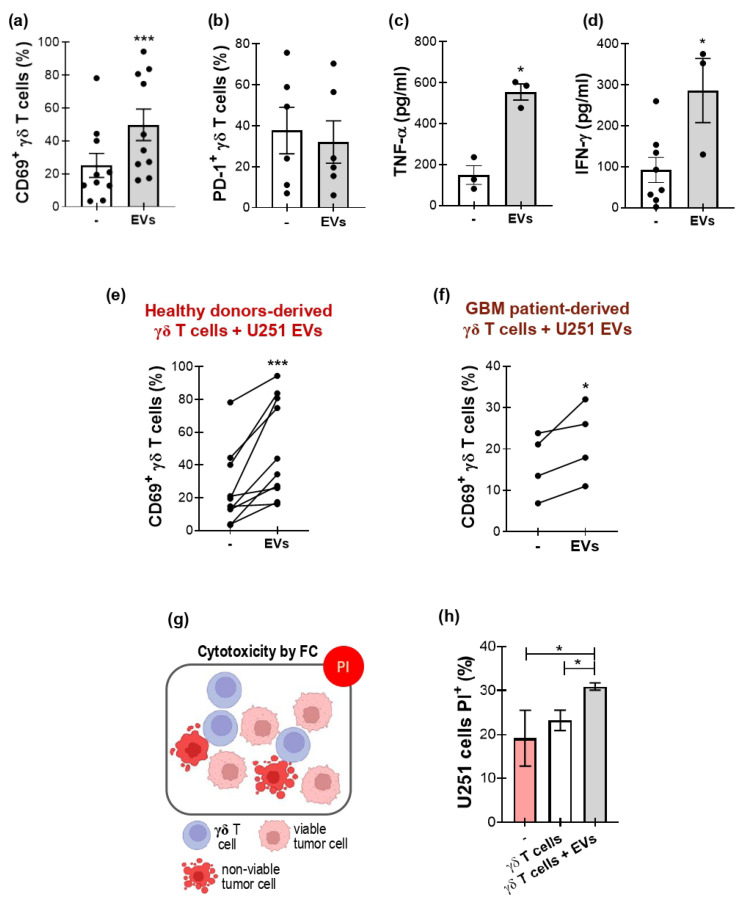
GBM-derived EVs modulate γδ T cells’ function. (**a**–**f**) γδ T cells were incubated ON with or without U251-derived EVs. Afterwards, the activation state of γδ T cells was analyzed by measuring the expression of CD69 and PD−1 by flow cytometry and the release of TNF-α and IFN-γ by ELISA. (**a**,**b**) Percentage of (**a**) CD69+ and (**b**) PD−1+ γδ T cells from healthy donors. One-tailed Wilcoxon test. (**c**,**d**) Concentration of (**c**) TNF-α and (**d**) IFN-γ in culture supernatants. One-tailed Mann–Whitney test. (**e**,**f**) Percentage of CD69+ γδ T cells from (**e**) healthy donors and (**f**) GBM patients. Black dots represents donor. One-tailed paired *t*-test. (**g**) Schematic diagram of propidium iodide (PI) labeling of apoptotic cells. (**h**) PI+ U251 cells after co-culture with γδ T cells, with (gray bar) or without (white bar) U251-derived EVs, measured by flow cytometry. Pink bar: U251 alone. Results are shown as the mean ± SEM. * *p* < 0.05, *** *p* < 0.001. Illustration (**b**) was designed in BioRender (https://app.biorender.com, accessed date 10 December 2025).

Based on these results, we employed that quantity of EVs throughout this study. Once the optimal conditions were set, we performed an ON incubation between γδ T cells and EVs. The activation state of γδ T cells was analyzed through the expression of CD69 and PD–1, and the production of the inflammatory cytokines. Figure 3 shows an increase in the percentage of expression of CD69 in γδ T cells (Figure 3a) and no changes in PD–1 expression (Figure 3b). According to the activation state triggered by EVs, there was an increase in the production of TNF-α (Figure 3c) and IFN-γ (Figure 3d). Notably, γδ T cells from GBM patients’ peripheral blood displayed the same behavior as cells from healthy donors upon exposure to U251-derived EVs (Figure 3e,f). The phenotypic and functional changes induced in γδ T lymphocytes by exposure to U251-derived EVs could be compatible with the acquisition of an antitumor and cytotoxic profile. To determine whether these EV-driven alterations translate into increased tumor-cell killing, we therefore evaluated γδ T-lymphocyte-mediated cytotoxicity against U251 target by propidium iodide staining and flow cytometry analysis (Figure 3g). Figure 3h shows that in the presence of EVs, γδ T cells showed an increased capacity to induce cytotoxicity in tumor cells, even higher compared to γδ T cells alone.

### 3.4. γδ T-Cell Activation by GBM-Derived EVs Is Dependent on the Presence of MICA/B on the EVs

**Figure 4:**
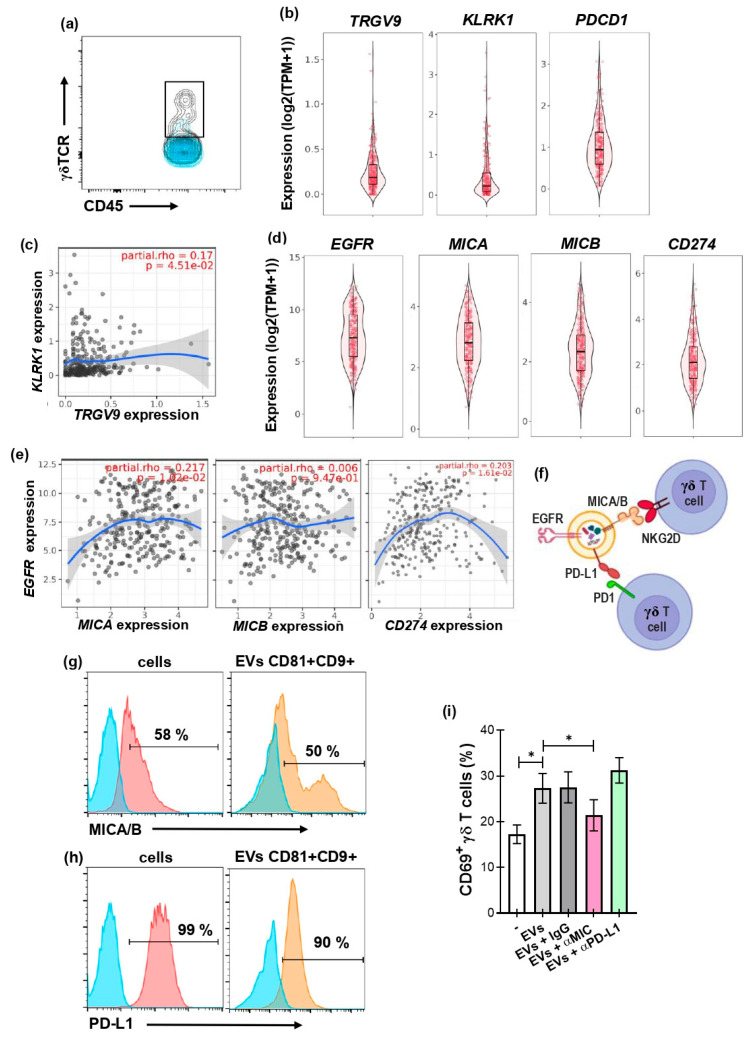
GBM-derived EVs modulate γδ T cells’ activation by a MIC-dependent mechanism. (**a**) Dot-plot of CD45+/γδTCR+ expression in a disaggregated GBM biopsy, analyzed by flow cytometry. (**b**–**h**) Transcript expression in GBM samples (from the TCGA database) was analyzed using TIMER 3.0. (**b**) Violin plots of *TRGV9* (**left panel**), *KLRK1* (**middle panel**), and *PDCD1* (**right panel**) expression in GBM samples. (**c**,**d**) Scatter plot showing the Spearman correlation between KLRK1 (**c**) and TRGV9 expression, adjusted for tumor purity. In (**c**) blue curve represents smoothed trend line and shaded area the 95% confidence interval. (**d**) Violin plots of *EGFR*, *MICA*, *MICB*, and *CD274* expression in tumor samples. (**e**) Scatter plot showing the Spearman correlation between *EGFR* and *MICA* (**left panel**), *MICB* (**middle panel**), and *CD274* (PD-L1) (**right panel**), adjusted for tumor purity. Blue curves show smoothed trend lines, with 95% confidence intervals indicated by the shaded area. Correlation coefficients and corresponding *p*-values are indicated. (**f**) Diagram of surface molecules present in U251-derived EVs and γδ T cells and their possible interactions. (**g**,**h**) Representative histograms showing the percentage of U251 cells (**left panel**) and CD9+/CD81+ U251 EVs (**right panel**) expressing MICA/B (**g**) or PD-L1 (**h**). Blue histogram represent FMO, pink represent cell staining and orange EVs staining. (**i**) Percentage of CD69+ γδ T cells when incubated ON with or without (white bar) U251-derived EVs and with IgG, anti-MIC, or anti-PD-L1 antibodies. Friedman and Dunn’s multiple comparisons tests. Results are shown as the mean ± SEM. * *p* < 0.05.

EV-associated ligands are particularly relevant for γδ T cells that recognize stress-induced molecules via germline encoded receptors and can rapidly exert effector programs including cytokine secretion and direct cytotoxicity in situ. Thus, EVs bearing ligands recognized by γδ T cells represent a plausible mechanism for tumor-driven modulation of γδ T-cell activity. Figure 4a shows the accumulation of γδ T cells in a disaggregated GBM biopsy, according to our previous in silico studies [36]. To go further in the study of molecules involved in γδ T-cell and GBM interactions, by using the public datasets TCGA and TIMER3.0 platform [40], we evaluate transcripts for gene encoding the main γδ TCR subtype represented in peripheral blood (Vγ9+ TCR): *TRGV9* (Figure 4b, left panel), NKG2D: *KLRK1* (Figure 4b, middle panel), and PD-1: PDCD1 (right panel) within the GBM tumor. A Spearman analysis showed a positive correlation between *TRGV9* and *KLRK1*, consistent with cytotoxic γδ T-cell presence (partial rho = 0.17, *p*-value = 0.045) (Figure 4c). In contrast, we found no correlation between *TRGV9* and *PDCD1* (Supplementary Figure S3). GBM samples also contain transcripts for *EGFR*, as well as *MICA*, *MICB*, and *CD274* (encoding gene of PD-L1), which play key roles in regulating the immune response against tumors [8] (Figure 4d). There is a positive correlation between *EGFR* and *MICA* (partial rho = 0.217, *p*-value = 0.01) (Figure 4e left panel), and with *CD274* (partial rho = 0.203, *p*-value = 0.016) (Figure 4e right panel), suggesting associations between tumor oncogenic phenotype and NKG2D and PD-1 ligands’ expression in the GBM microenvironment. Of note, this analysis was performed using tumor databases rather than databases of EVs because no information was available on MICA/B expression in GBM-derived EVs.

With these in silico results in mind, we therefore sought to determine whether U251-derived EVs express MICA/B and PD−L1 (Figure 4f). Interestingly, the isolated EVs express MICA/B (Figure 4g) and PD−L1 (Figure 4h), like their source cells. Blocking experiments showed that the activation of γδ T cells was MIC-dependent since the preincubation of EVs with neutralizing antibodies markedly reduced γδ T-cell activation, supporting a role for MIC as an important activating pathway. In contrast, blockage of PD−L1 on U251 EVs did not alter γδ T-cell activation under the conditions tested, suggesting that PD−L1 is not a dominant regulator of γδ T-cell responses to U251 EVs in our model system (Figure 4i).

## 4. Discussion

In this work, we characterized EVs isolated from the GBM cell line U251 by TEM, NTA, spectral flow cytometry, and Western blot and showed that the preparation was enriched in medium/large EVs. These vesicles contain canonical tetraspanins (CD9, CD63, CD81), tumor-associated proteins such as EGFR, the immune modulator molecule PD−L1, and stress ligands MICA/B, and they also contain nucleic acids. The combined morphological, biophysical, and molecular evidence supports the classification of the isolated material as bona fide EVs. Of note, complete removal of non-EV material is not achievable with current methods, and although separation techniques differ in effectiveness, none reliably exclude all co-isolated contaminants such as protein aggregates or apoptotic bodies. Consequently, empirical characterization is essential to evaluate the purity of EV preparations. In our hands, TEM revealed the characteristic morphology and intact membranes expected for EVs, supporting exclusion of non-vesicular contaminants. Cells recovered after supernatant collection showed high viability, which argues against substantial contamination by apoptotic bodies. Because co-isolated components can influence biological activity, method selection must balance yield and purity and be tailored to each study’s goals. In this work, we focused on medium/large EVs for three reasons: (1) it has been described that they are more abundant than smaller EVs [37]; (2) they carry a larger proportion of mRNAs reflecting mutational status and gene amplification, making them a more representative peripheral readout of source-cell content [42]; and (3) their size facilitates isolation and downstream molecular analyses. Confocal imaging and color spatial and intensity heterogeneities revealed frequent and dynamic contacts between PKH26-labeled U251 EVs and primary human γδ T cells, in which vesicles are observed in states related to surface interaction and uptake. The kinetics of EVs interaction suggest that vesicle engagement is dynamic and influenced by the activation status of the lymphocyte. EV uptake increased over time and was quite heterogeneous at the single-cell level, consistent with activation-driven modulation of EVs binding and internalization. Of note, orthogonal projections and three-dimensional construction confirmed that a substantial fraction of the PKH26 signal localized beneath the plasma membrane, indicating genuine uptake rather than artifactual surface overlap. Because internalized EVs can deliver membrane-bound ligands and soluble cargo to recipient cells, these interactions provide a plausible mechanism by which GBM EVs modulate γδ T-cell activation and effector function, as explored in subsequent functional assays we have done. Nevertheless, the relative contributions of membrane receptor engagement versus intracellular signaling after EV internalization remain to be dissected.

Exposure to U251-derived EVs induced an activation program in γδ T cells that involved the upregulation of CD69, increased secretion of IFN-γ and TNF-α, and enhanced cytotoxicity against U251 targets. This is consistent with our previous finding that factors released by GBM cells promote a Th1-like functional profile [36]. Remarkably, the phenotypic changes were observed in γδ T cells from healthy donors and from GBM patients, indicating that the EV-driven response is robust across donor sources and that γδ T cells from patients are able to respond to GBM-derived EVs. Of note, while in vitro assays are valuable for demonstrating direct interactions and dissecting mechanisms under controlled conditions, they may not fully reproduce certain in vivo features; therefore, further studies using patient-derived cells and EVs are needed to confirm the translational relevance of these results. Blocking experiments implicate MIC on the EV surface as the principal mediator of γδ T-cell activation. These data argue for a causal role of EV-associated MICA/B in triggering γδ T-cell effector programs. Although PD−L1 was detectable on U251 EVs, neutralization of this immune checkpoint molecule did not measurably alter γδ T-cell activation in our in vitro assays. This suggests that, within the constraints of our model system and experimental conditions, the activating signal delivered by MICA/B predominates over PD−L1-mediated inhibition. Nevertheless, PD−L1 may exert context-dependent effects in vivo or in combination with other suppressive factors present in the tumor microenvironment; the coexistence of activating and inhibitory molecules on tumor EVs highlights the complex and potentially tunable nature of EV-mediated immune modulation. Thus, these negative results do not exclude context-dependent roles for PD−L1 in other settings, but they indicate that MICA/B is the primary determinant of EV-mediated γδ T-cell stimulation in our model. The MICA/B participation identifies a specific mechanism by which tumor EVs influence γδ T-cell responses and suggests potential strategies to enhance γδ T-cell-mediated antitumor activity by targeting EV-associated stress ligands. Interestingly, the bioinformatic analysis has supported the results we obtained in vitro, showing a positive correlation between TRGV9 and NKG2D and MICA/B with EGFR in GBM tumors. Our results provide a mechanistic proof of principle that GBM-derived EVs carry MICA/B and activate human γδ T cells via the MICA–NKG2D axis. However, translation to the clinic will require validation with patient-derived EVs because they may be biologically heterogeneous and differ in marker expression and immunomodulatory potency. U251-derived EVs may not reflect tumor diversity and inter-patient variability. Future work should validate these observations using EVs from primary GBM specimens and patient plasma, test the in vivo relevance of EV-mediated γδ T-cell activation, and distinguish surface signaling from cargo-dependent intracellular effects using targeted perturbations of endocytic pathways and compartment-specific reporters.

In summary, U251-derived EVs are medium/large particles that carry tumor and stress markers, physically interact with and are internalized by γδ T cells, and drive a functional activation program that enhances cytokine production and cytotoxicity. The stimulatory effect is mediated primarily by EVs carrying MICA/B. However, this activation and differentiation can occur in an inappropriate spatial and temporal context within the tumor, and the resulting γδ T lymphocytes are functionally misdirected and so unable to mount an effective cytotoxic response against tumor cells. Instead of promoting tumor clearance, these activated lymphocytes may contribute to a dysfunctional immune milieu that permits tumor persistence and progression. These results identify a specific EV-mediated pathway by which GBM cells can engage γδ T lymphocytes and suggest avenues to harness or modulate EV–ligand interactions for immunotherapeutic benefit.

## 5. Conclusions

Our study shows that U251-derived EVs carry MICA/B, are taken up by human γδ T cells, and activate them via the MICA–NKG2D axis—increasing CD69, IFN-γ, TNF-α, and cytotoxicity—while MICA/B blockade attenuates this response.

Although these results require validation with patient-derived EVs and in vivo models, they identify MICA/B on GBM-derived EVs as a potential target to enhance γδ T-cell-based antitumor immunotherapies.

## Figures and Tables

**Table 1:** Characterization of EVs derived from GBM cell line U251. EVs were obtained from confluent monolayers of 6 × 10^6^ U251 cells after differential centrifugations. They were resuspended either in PBS for NTA analysis or in TRIzol for RNA quantification. Results are shown as mean ± SEM of the diameter, Z potential, particle concentration (n = 4), and RNA concentration of EV samples (n = 3).

Size (nm)	Z Potential (mV)	Particles/mL	RNA (ng)
184.0 ± 6.1	−46.2 ± 1.4	2.1 × 10^11^ ± 0.3 × 10^11^	345.7 ± 32.9

## Data Availability

The datasets generated for this study are available on request to the corresponding author. The datasets employed for the transcriptomic meta-analysis in GBM samples were obtained from The Cancer Genome Atlas (TCGA): https://portal.gdc.cancer.gov (accessed on 17 December 2025).

## Supplementary Materials

The following supporting information can be downloaded at https://www.mdpi.com/article/10.3390/biology15030275/s1. Table S1: Donors and patients. Figure S1: Assay controls for GBM-derived EVs’ flow cytometry. Figure S2: U251−derived EVs’ titration. Figure S3: Transcript expression in GBM samples.

## Abbreviations

CTLA–4: cytotoxic T-lymphocyte-associated protein 4; EGFR: epidermal growth factor receptor; EVs: extracellular vesicles; FASL: FAS ligand; GBM: glioblastoma; HMBPP: (*E*)-4-hydroxy-3-methyl-but-2-enyl pyrophosphate; IFN-γ: interferon gamma; KLRK1: killer cell lectin-like receptor subfamily K member 1; MHC: major histocompatibility complex; MICA/B: MHC class I chain-related A/B; NTA: nanoparticle tracking analysis; NKG2D: natural killer group 2 member D; PD–1: programmed cell death 1; PD–L1: programmed death-ligand 1; TCGA: The Cancer Genome Atlas; TCR: T-cell receptor; TEM: transmission electron microscopy; Th1: T helper 1; TNF-α: tumor necrosis factor alpha; TRGV9: T-cell receptor gamma variable 9.
