# Supplementary material for: Small Messengers: Glioblastoma-Derived Extracellular Vesicles Modulate γδ T Lymphocytes Through a MIC-Dependent Mechanism
Source: Biology (Basel). 2026 Feb 3;15(3):275. doi: 10.3390/biology15030275 (PMC12896963; doi:10.3390/biology15030275)
Supplement: Supplementary file 1 [file biology-15-00275-s001.zip › biology-4090126-supplementary.pdf]

Supplementary material

Table S1: Donors and patients

1. Healthy blood donors:

**Number**	22 samples
**Sex distribution**	Female: 6, Male: 16
**Average age**	41.3 years old
**Inclusion criteria**	1) The donor of blood must be between 21 and 65 years old.<br>2) Hg ≥ 12.5 g/dl<br>3) Hematocrit ≥ 38%.<br>4) Beats per minute between 50 and 100.<br>5) Systolic blood pressure, between 90 and 180 mmHg. Diastolic pressure between 60 and 100 mmHg. People who have no other health considerations and who are taking medications to control their blood pressure can donate blood if their blood pressure is within acceptable limits.<br>6) Body weight equal to or greater than 50 kg.
**Exclusion criteria**	1) Having had viral hepatitis after age 10, other than Hepatitis A.<br>2) Have or have had clinical or laboratory evidence of infections by *Tripanosoma cruzi*, HIV, HTLV, HCV, and/or HBV.<br>3) Injecting drug users not prescribed by doctors.<br>4) Persons who suffer from Hemophilia or are hemodialysis or periodically receive transfusions of blood, its components or derivatives.<br>5) Have had repeatedly suffered from syphilis or gonorrhea. Those potential donors who report having suffered a single episode with complete and adequate treatment may be included in a readmission protocol with a medical interview and a negative screening test.<br>6) Are at risk for Creutzfeldt-Jakob disease, or its variant. Have a family history of the disease.<br>7) Have received pituitary hormone of human origin between 1958 and 1966.<br>8) Has received a brain tissue or membrane transplant.<br>9) Have been reside for more than one year (adding all the periods of stay) in the United Kingdom during the period from 1980 to 1996, or in countries that have had foci of infection by CJ V.<br>10) Not having suffered, or have been at risk of contracting infections liable to be transmitted by transfusion (ITT). Information related to travel or stay in areas with a high prevalence of endemic ITTs (leishmania, borrelia, dengue, Variant of the agent of Creutzfeldt-Jakob disease, West Nile virus, among others) should be collected.<br>11) Pregnancy contraindicates donation. Women will be excluded for 6 weeks after a normal delivery, 12 months after a caesarean section or an abortion followed by an evacuation curettage. It is recommended that nursing mothers do not donate blood.

	12) People who have undergone endoscopies will be excluded for a period of 6 months. Regarding laparoscopies and surgeries, a medical evaluation is necessary before accepting as a donor. When it comes to uncomplicated surgeries, it should be postponed for six months after the intervention. The deferral should be extended to 12 months if the person received transfusions.<br>14) At the time of evaluation, there were no signs or symptoms of fever.<br>13) They are disqualified for 12 months (temporarily) as donors of blood or blood components, those who have received tattoos, non-sterile skin piercing and/or acupuncture or suffered occupational accidents with exposure to blood or secretions (punctures or contact with them through mucous membranes or wounds).<br>14) Potential donors who have received blood transfusions, components or blood derivatives must be deferred for twelve months.

2. GBM patients:

**Number**	4 samples
**Average age**	54.7 years old
**Sex distribution**	Female: 2, Male: 2
**Inclusion criteria**	1) Age ≥ 21 years.<br>2) No documented prior oncologic disease.<br>3) No documented prior immunological disease.<br>4) Brain magnetic resonance imaging with findings suggestive of glioblastoma.<br>5) Documented written informed consent for the collection and use of surgical/hospitalization samples specific to this study.
**Exclusion criteria**	1) Not meeting the inclusion criteria.<br>2) Not consenting to the use of samples obtained during the surgical procedure or hospitalization.

Figure S1

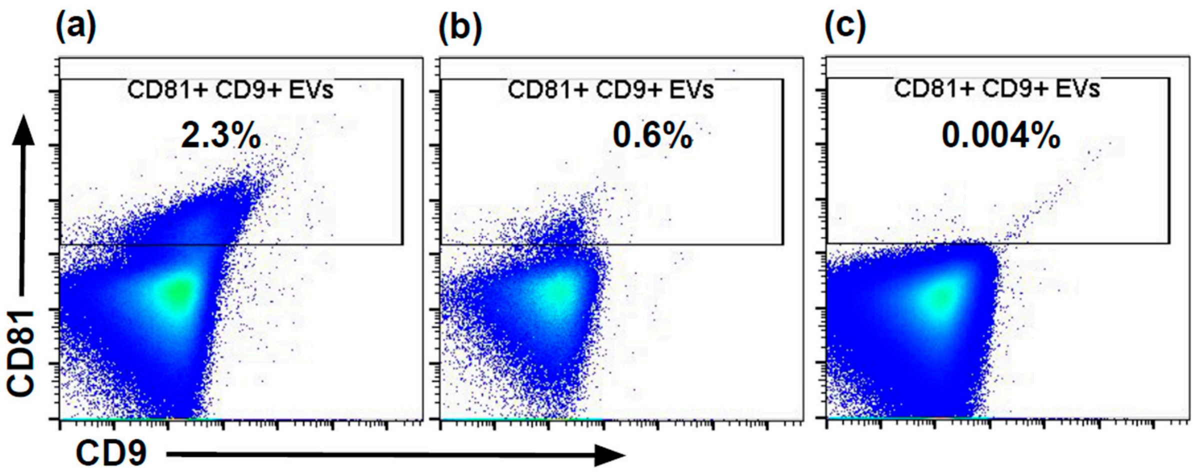

Fig. S1. Assay controls for GBM-derived EVs' flow cytometry. The material isolated from the supernatants of the GBM cell line U251 via differential centrifugation was stained with fluorochrome−conjugated antibodies and analyzed by full-spectrum flow cytometry. **(a−c)** CD81−FITC fluorescence intensity versus yuCD9−PerCP/Cy5.5 fluorescence intensity of a stained sample **(a)** before and **(b)** after being treated with Triton X−100 for 5 min. The EVs gate shows stained particles (CD81+/CD9+ or only CD81+) from background noise and unstained particles. **(c)** Dot plot of the buffer with reagents only.

Figure S2

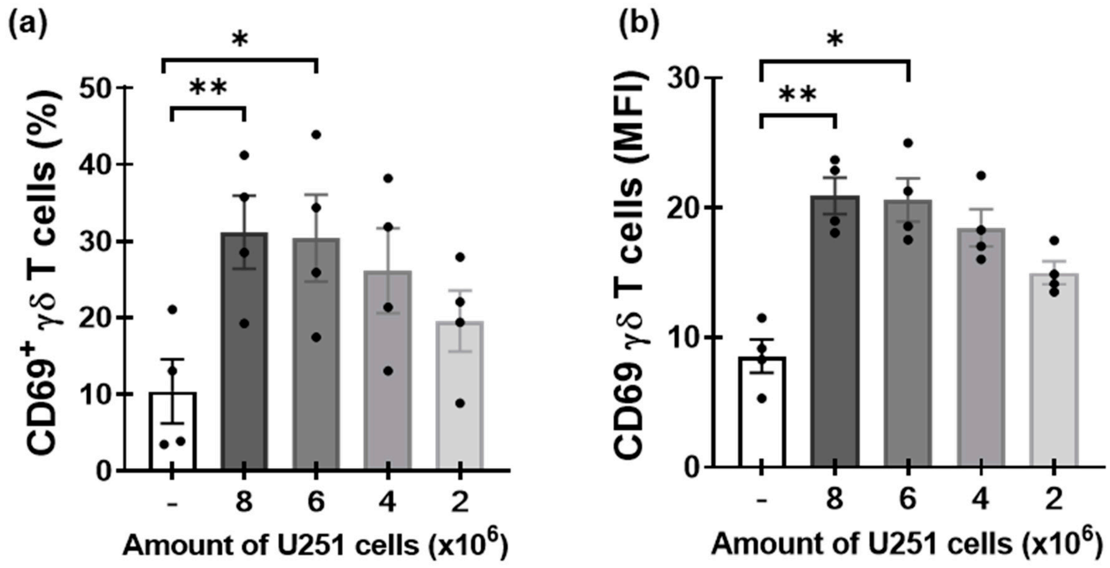

Fig. S2. U251−derived EVs' titration. (a-b) γδ T cells were incubated ON with or without EVs

samples from different amounts of U251 cells: 2 to $8x10^6$. Afterwards, the activation state of γδ T cells was analyzed by measuring the expression of CD69 by flow cytometry. **(a)** Percentage of CD69+ γδ T cells of healthy donors. **(b)** Median fluorescent intensity of CD69 expression in γδ T cells. Friedman and Dunn's multiple comparisons tests. Results are shown as the mean ± SEM. **p*<0.05, ***p*<0.01.

Figure S3

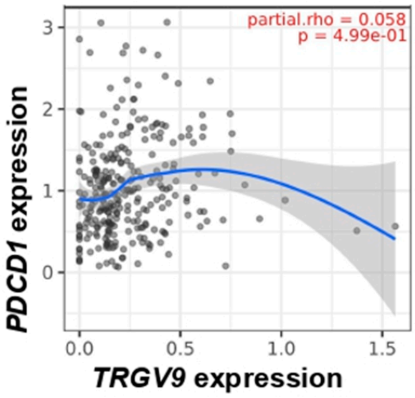

Figure S3: Transcript expression in GBM samples. Database from the TCGA was analyzed using TIMER 3.0. Scatter plot shows the Spearman correlation between PDCD1 and TRGV9 expression, adjusted for tumor purity. The blue curve represents the smoothed trend line and shaded area the 95% confidence interval.
